# Supplementary material for: Single-cell and spatial multiomic inference of gene regulatory networks using SCRIPro
Source: Bioinformatics. 2024 Jul 18;40(7):btae466. doi: 10.1093/bioinformatics/btae466 (PMC11288411; doi:10.1093/bioinformatics/btae466)
Supplement: btae466_Supplementary_Data [file btae466_supplementary_data.zip › Supplementary_Figures.docx]

**Supplementary figures**

**
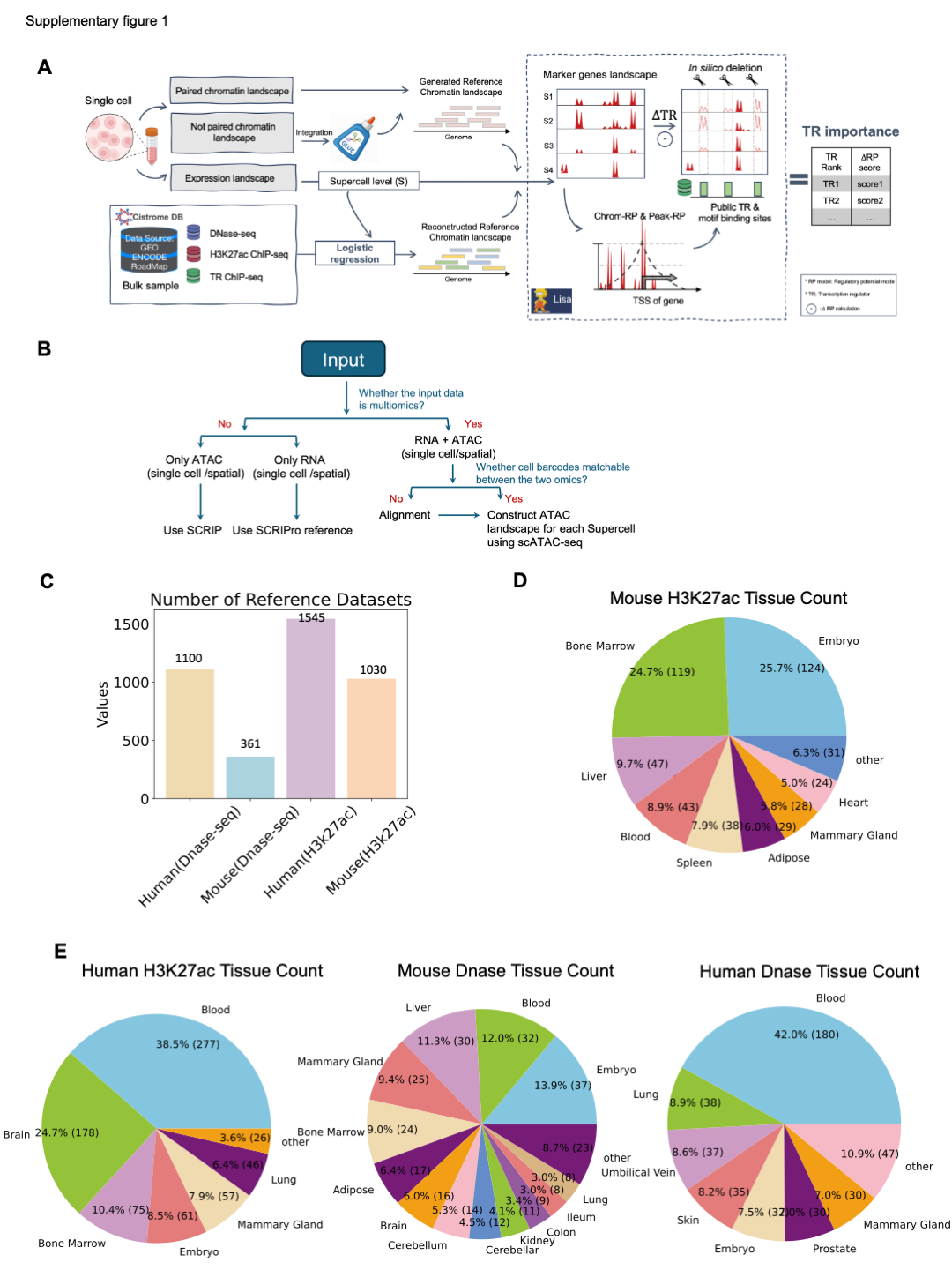
**

**Fig. S1 Details algorithm schematic of SCRIPro and statistics of chromatin reference in human and mouse, Related to Fig.1.**

1. The input data is divided into two parts: single-cell RNA-seq and chromatin accessibility data, which can be single-cell matched or DNase-seq and H3K27ac ChIP-seq experiments bulk data collected from the Cistrome DB. Then SCRIPro construct a marker genes landscape for each supercell. RP model is used to calculate the RP (chrom-RP and peak-RP) distribution score for each gene, taking into account nearby DNase-seq and H3K27ac ChIP-seq peaks. The RP model is utilized to calculate the RP distribution score for each gene, incorporating peaks from nearby DNase-seq and H3K27ac ChIP-seq data. Subsequently, in silico deletion of TR binding sites from the reference database is conducted to perform a Wilcoxon test between the two distributions, resulting in a ∆RP score for each TR. This facilitates the prioritization of TRs by their activity score within each supercell.
2. Processing logic diagram of SCRIPro:
3. For transcriptomic-only data, we employ strategies similar to those used in LISA to reconstruct chromatin landscapes from bulk DNase/H3K27ac references.
4. For epigenomic-only data, SCRIP is utilized to infer potential regulators.
5. For multi-omics data, we perform alignment and use matched or paired epigenome datasets to construct chromatin landscapes.
6. Number of reference datasets of DNase-seq and H3K27ac in both human and mouse.

D-E. Pie chart of H3K27ac and DNase-seq tissue count percentage in mouse and human.


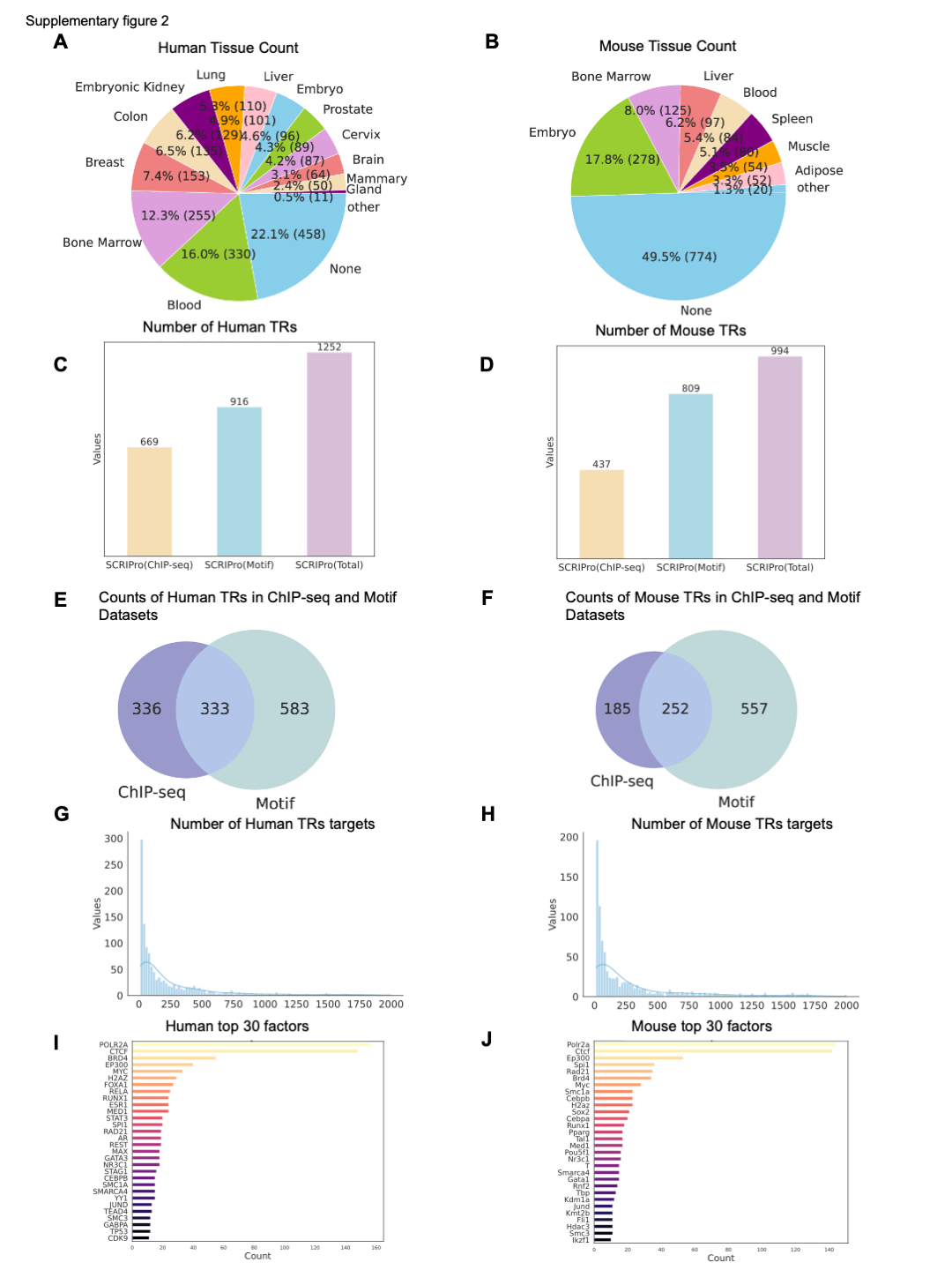


**Fig. S2 Statistics of TR reference in human and mouse, Related to Fig.1.**

1. Percentage distribution of human tissue ChIP-seq datasets used as part of SCRIPro references (669 in total), collected from the Cistrome Data Browser.
2. Percentage distribution of mouse tissue ChIP-seq datasets used as part of SCRIPro references (437 in total), collected from the Cistrome Data Browser.
3. Bar plot showing the statistics of human reference. Left: the number of ChIP-seq datasets (669); Middle: the number of motifs (916); Right: the total number in human SCRIPro references (1252).
4. Bar plot showing the statistics of mouse reference. Left: the number of ChIP-seq datasets (437); Middle: the number of motifs (809); Right: the total number in mouse SCRIPro references (994).
5. Venn diagram illustrating the number of ChIP-seq and motif datasets individually and their overlap (333) in human TRs.
6. Venn diagram illustrating the number of ChIP-seq and motif datasets individually and their overlap (252) in mouse TRs.
7. The number of targets with a regulatory potential greater than 5 in each set of the human ChIP-seq reference.
8. The number of targets with a regulatory potential greater than 5 in each set of the mouse ChIP-seq reference.
9. Bar plot displaying the counts of the top 30 factors in human PBMC dataset.
10. Bar plot displaying the counts of the top 30 factors in mouse hair follicles dataset.


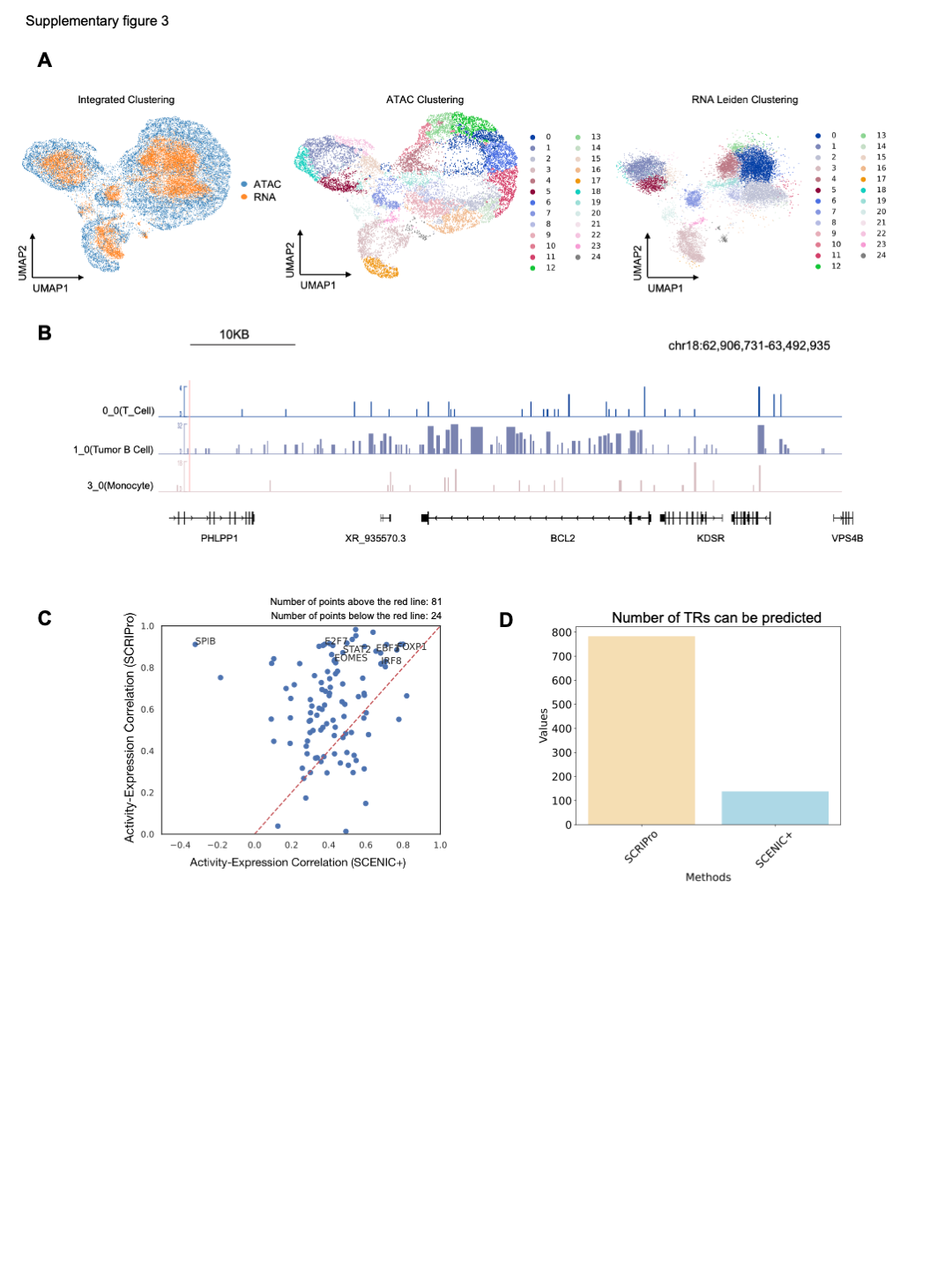


**Fig. S3 Performance comparison between SCRIPro and SCENIC+ on human B-cell lymphoma data, Related to Fig.3.**

1. UMAP of multiome data type after integration by SCRIPro.
2. IGV showed celltype specific TR BCL2 identified by SCRIPro.
3. Correlation plot showing the relationship between activity scores and expression for each TR shared between SCRIPro and SCENIC+. X-axis: The activity-expression correlation in SCENIC+. Y-axis: The activity-expression correlation in SCRIPro.
4. Bar plot showing the number of TRs predicted by SCRIPro (783) and SCENIC+ (138) respectively. Threshold for SCRIPro: TR appears in more than 100 cells and greater than 0.1. Threshold for SCENIC+: no cutoff.


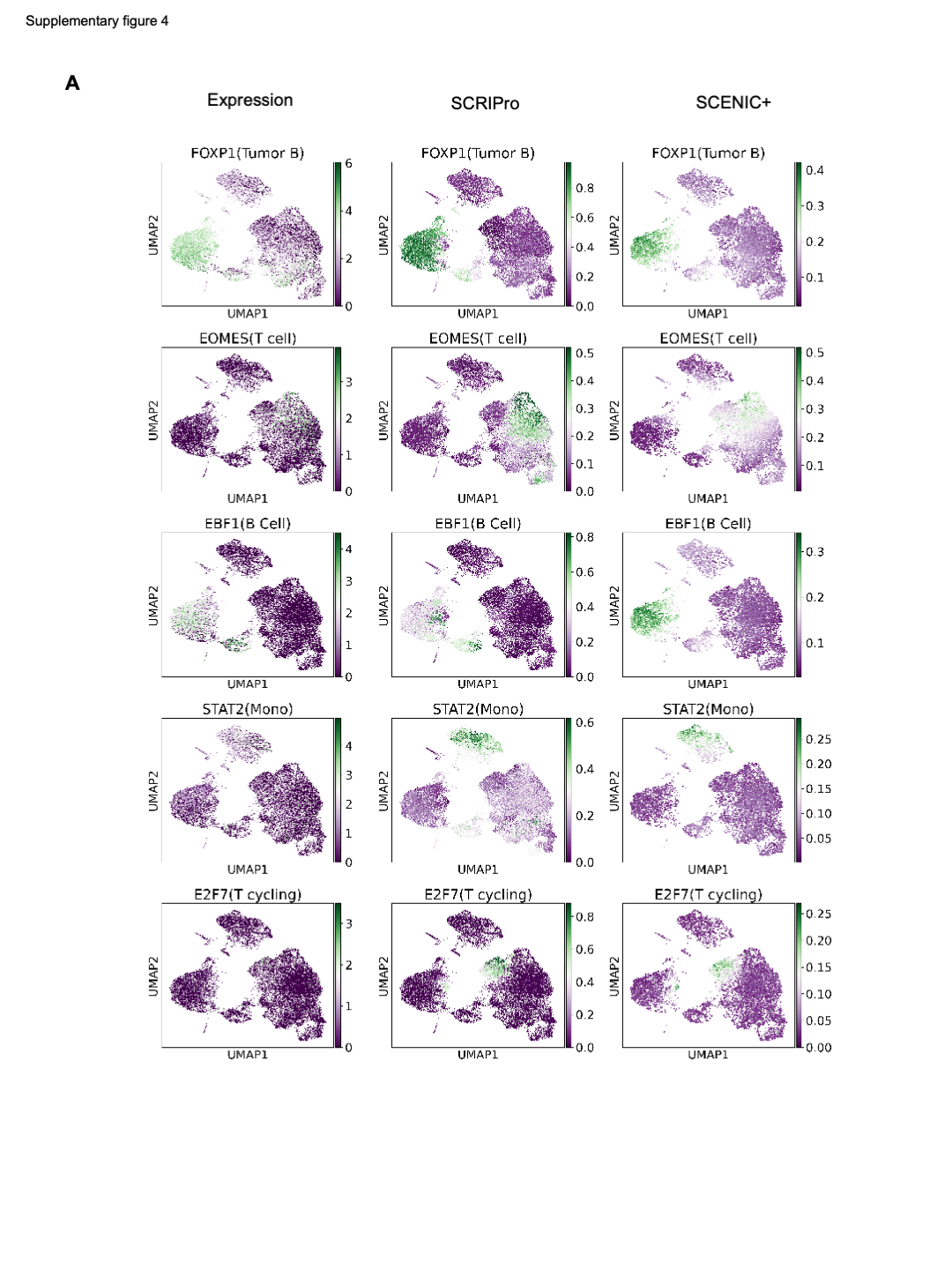


**Fig. S4 Performance comparison between SCRIPro and SCENIC+ on human B-cell lymphoma data, Related to Fig.3.**

1. UMAP distribution of marker TRs across different cell types. FOXP1 (Tumor B cell), EOMES (T cell), EBF1 (B cell), STAT2 (Monocyte), E2F7 (T cycling).


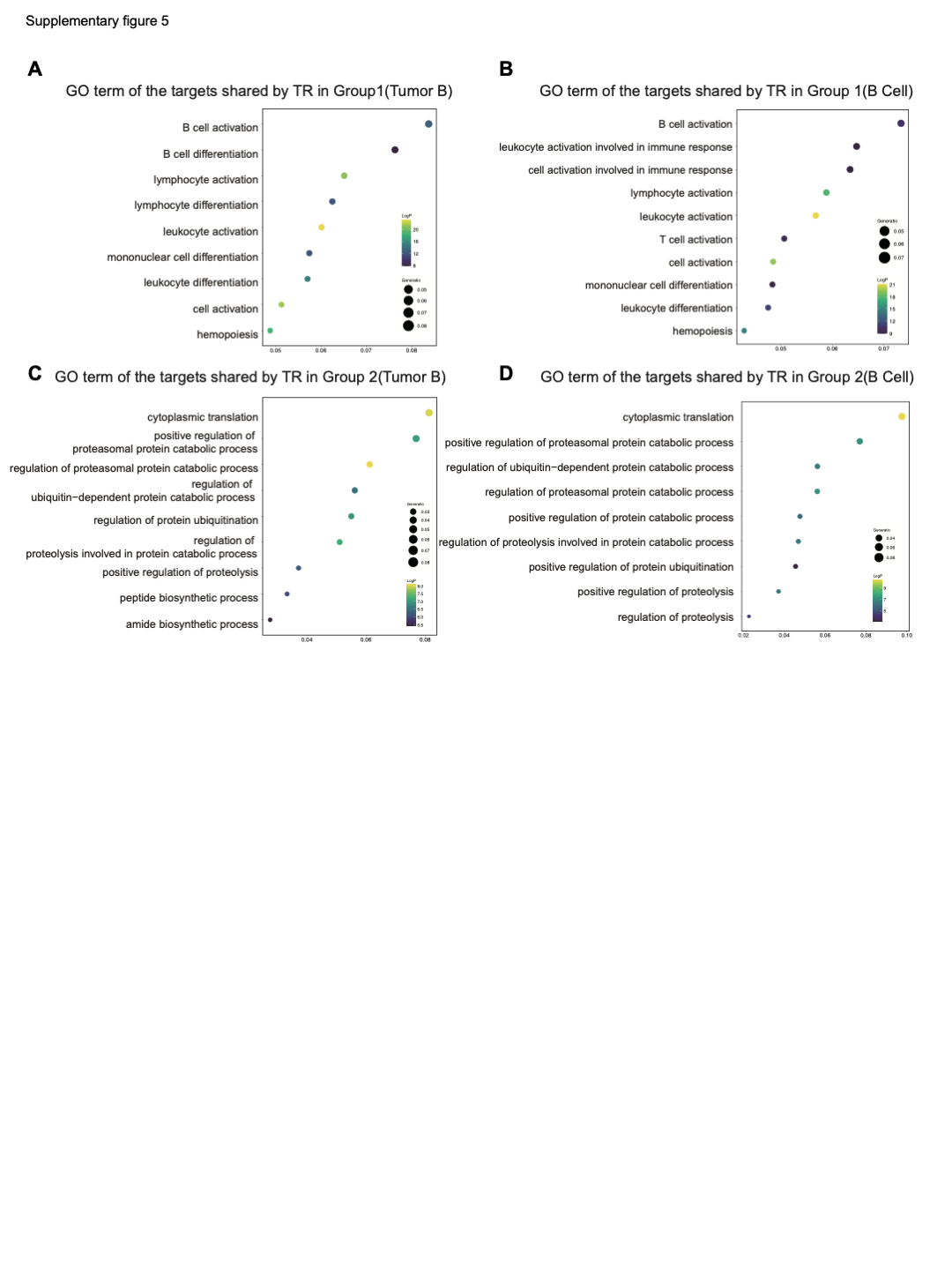


**Fig. S5 GO terms for shared targets of TRs in Group 1 and 2 within Tumor B cell type and B cell type, Related to Fig.3.**

1. GO term of the targets shared by TRs in Group 1 in tumor B cell type.
2. GO term of the targets shared by TRs in Group 1 in B cell type.
3. GO term of the targets shared by TRs in Group 2 in tumor B cell type.
4. GO term of the targets shared by TRs in Group 2 in B cell type.


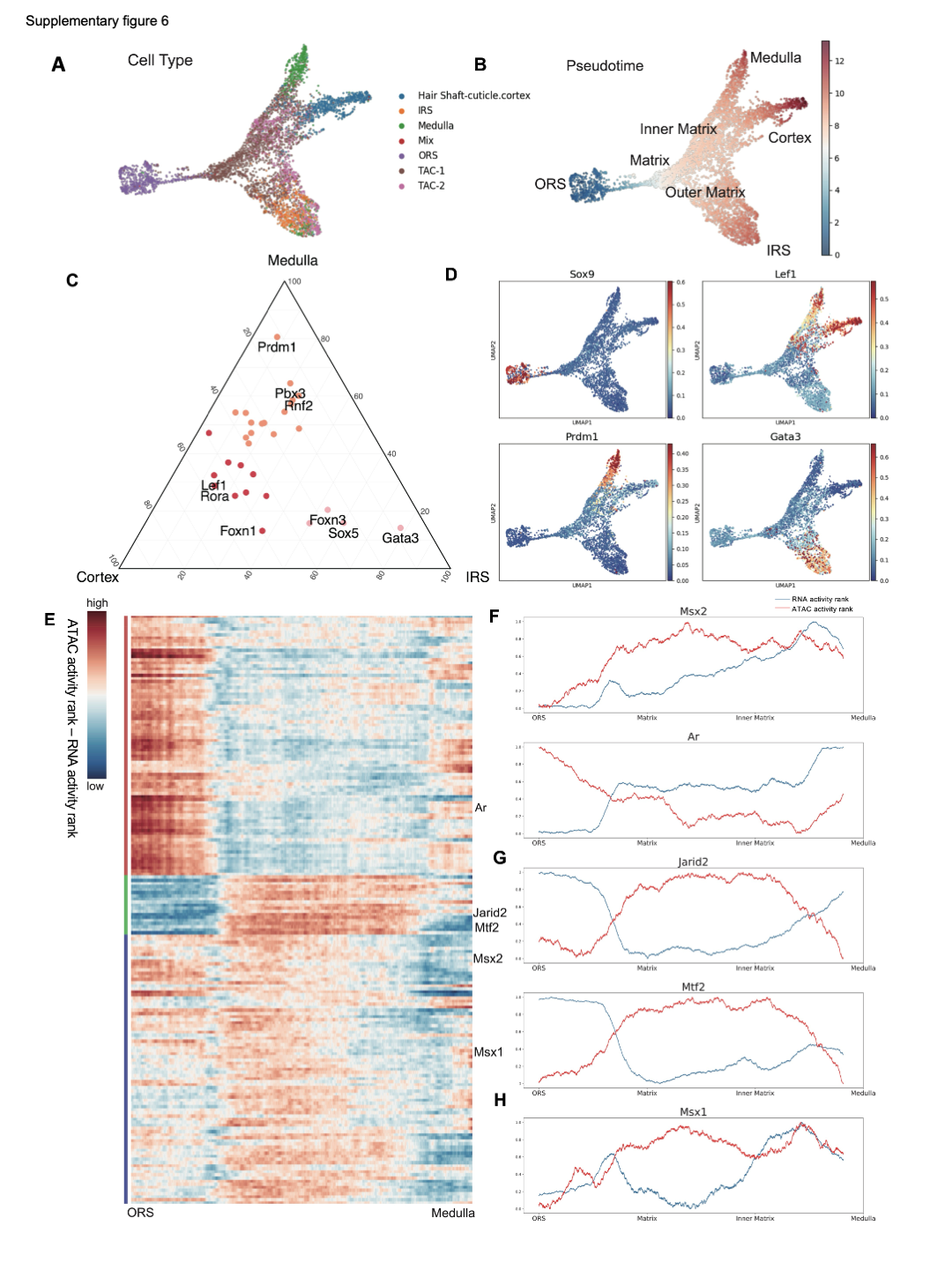


**Fig. S6 SCRIPro reveals epigenetic priming effect in the mouse hair follicle development SHARE-seq data.**

1. UMAP showing 7 cell types during hair follicle development. IRS: Inner Root Sheath. ORS: Outer Root Sheath.
2. Pseudotime UMAP plot illustrating the developmental trajectory of hair follicle structure, with different nodes representing various tissue types.
3. Ternary plot showing TRs enriched in Medulla, Cortex, and IRS tissue types, respectively. Colors represent different tissue types, and numbers indicate the relative enrichment level.
4. UMAP showing the distribution of TR activity of Sox9, Lef1, Prdm1, and Gata3.
5. Heatmap illustrating the clustering of all TRs along the developmental trajectory from ORS to medulla, based on the difference in ATAC and RNA values. Left: 3 groups identified by the heatmap clustering. Infer score: The difference in rank values of all TRs between SCRIP and SCRIPro. Red indicates ATAC activity rank – RNA activity rank > 0, while blue indicates ATAC activity rank – RNA activity rank < 0.

F-H. RNA activity score and ATAC activity score of group 1-3 (Src, Nfyb, Jarid2, Mtf2 and Msx1) from ORS to medulla.


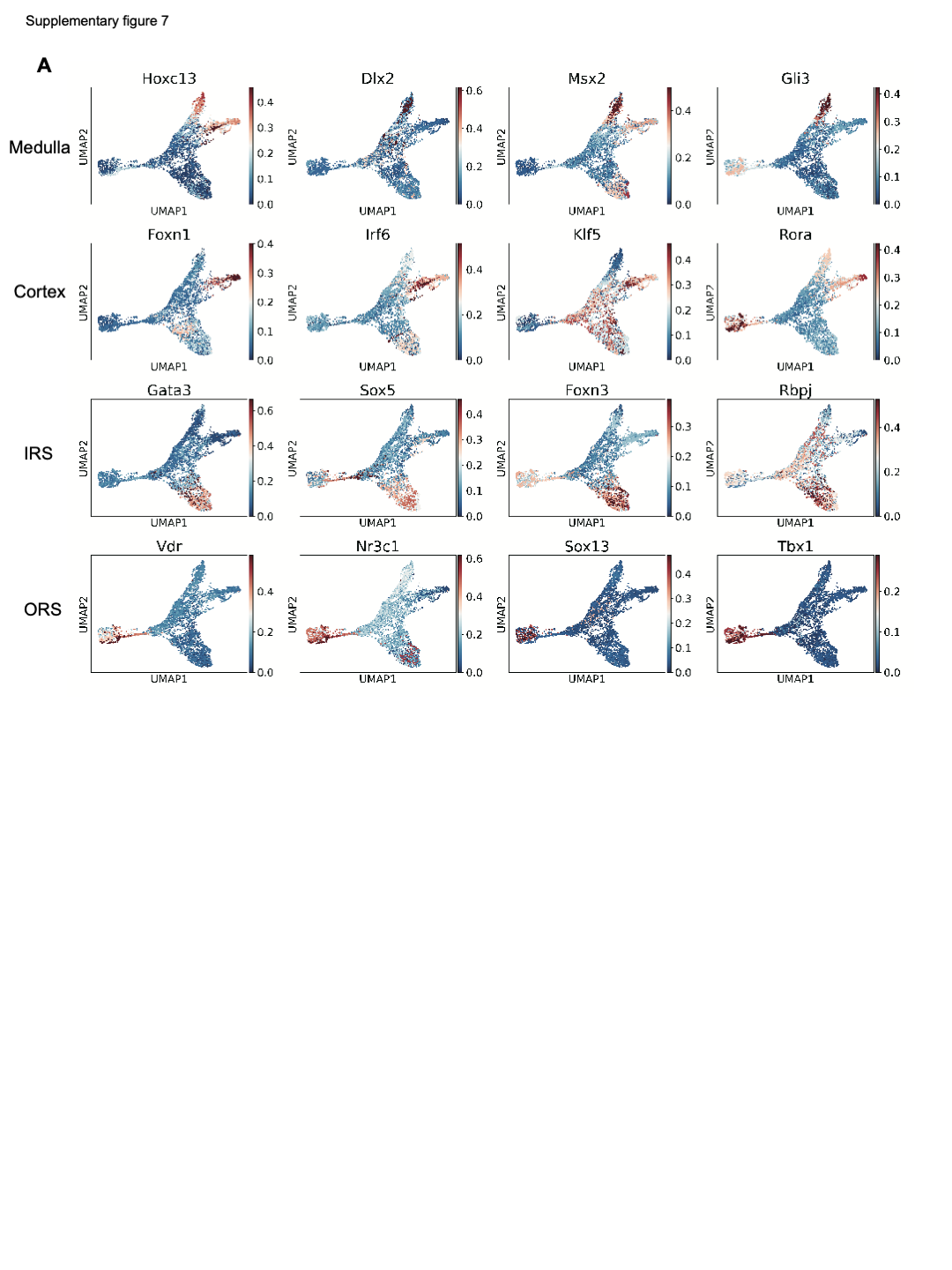


**Fig. S7 UMAP distribution of TRs enriched in different cell types in mouse hair follicle development data, Related to Fig. S6.**


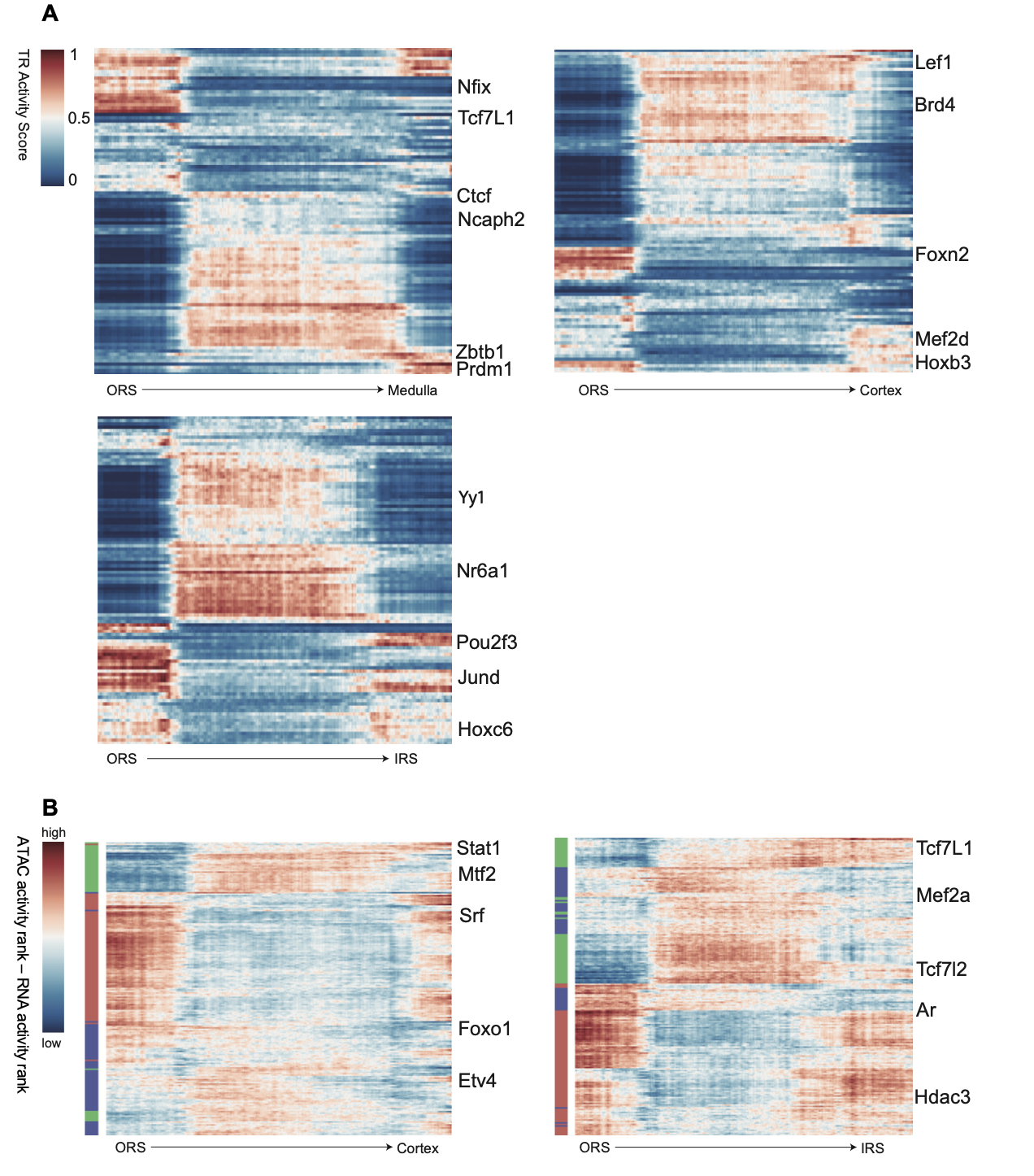
 **Fig. S8 Heatmap of TRs clustering along different pseudotime trajectories in mouse hair follicle development data, Related to Fig. S6.**

1. Heatmap clustering of TRs along ORS-Medulla, ORS-Cortex and ORS-IRS developmental trajectories based on normalized TR activity score scale. Key TRs are labeled on the right.
2. Heatmap clustering of TRs along ORS-Cortex and ORS-IRS developmental trajectories based on normalized ATAC activity rank – RNA activity rank value scale. Key TRs are labeled on the right.


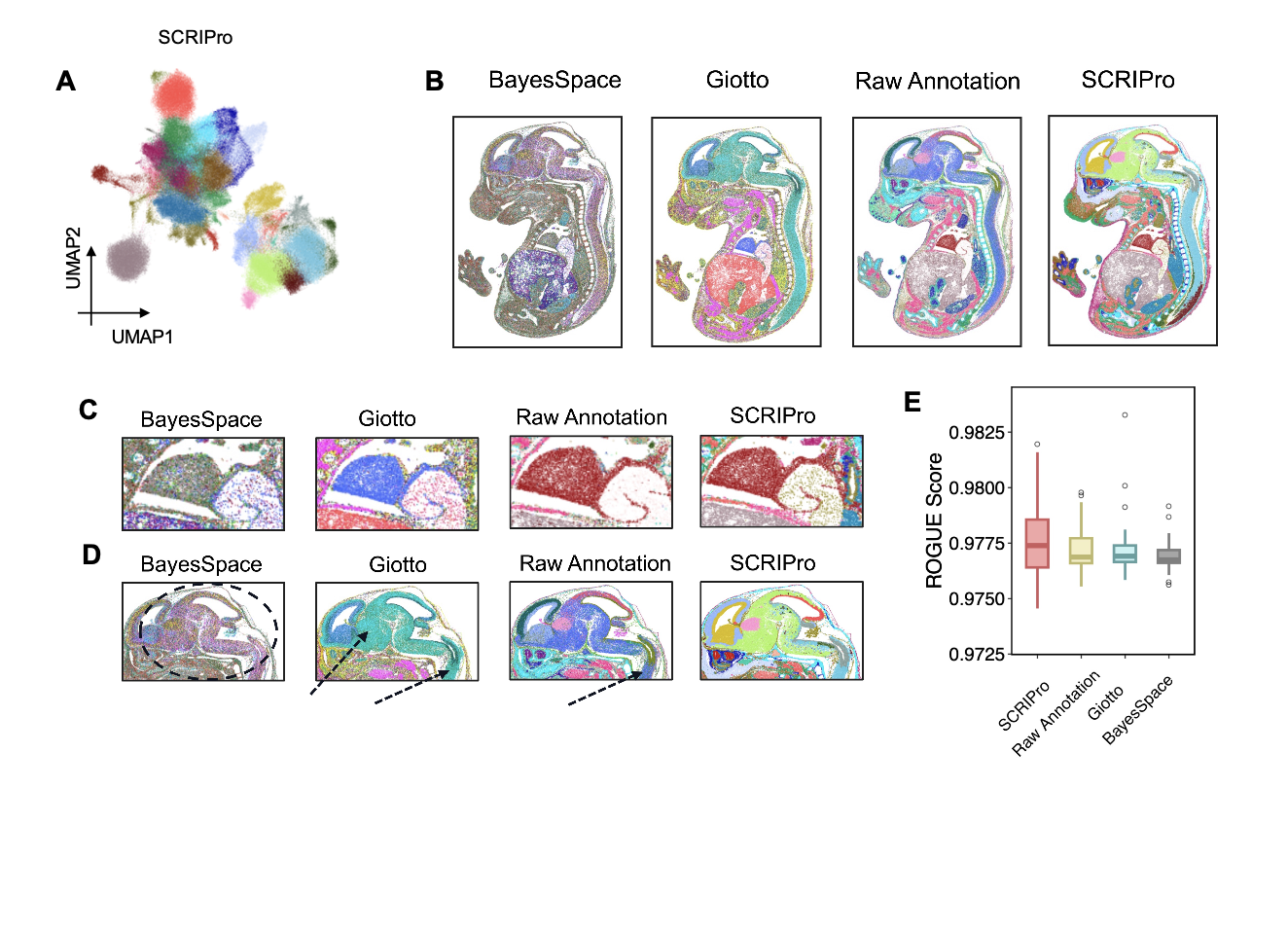


**Fig. S9 Performance comparison between SCRIPro and other spatial clustering methods in predicting TRs using spatial data, Related to Fig.4.**

1. The UMAP of E16.5 Stereo-seq mouse embryo data was generated using SCRIPro.
2. The spatial clustering of BayesSpace, Giotto, Raw annotation(using Squidpy) and SCRIPro.
3. The spatial clustering of BayesSpace, Giotto, Raw annotation(using Squidpy) and SCRIPro in heart, mid/hindbrain and part of spinal cord region.
4. ROGUE score of BayesSpace, Giotto, Raw annotation(using Squidpy) and SCRIPro.


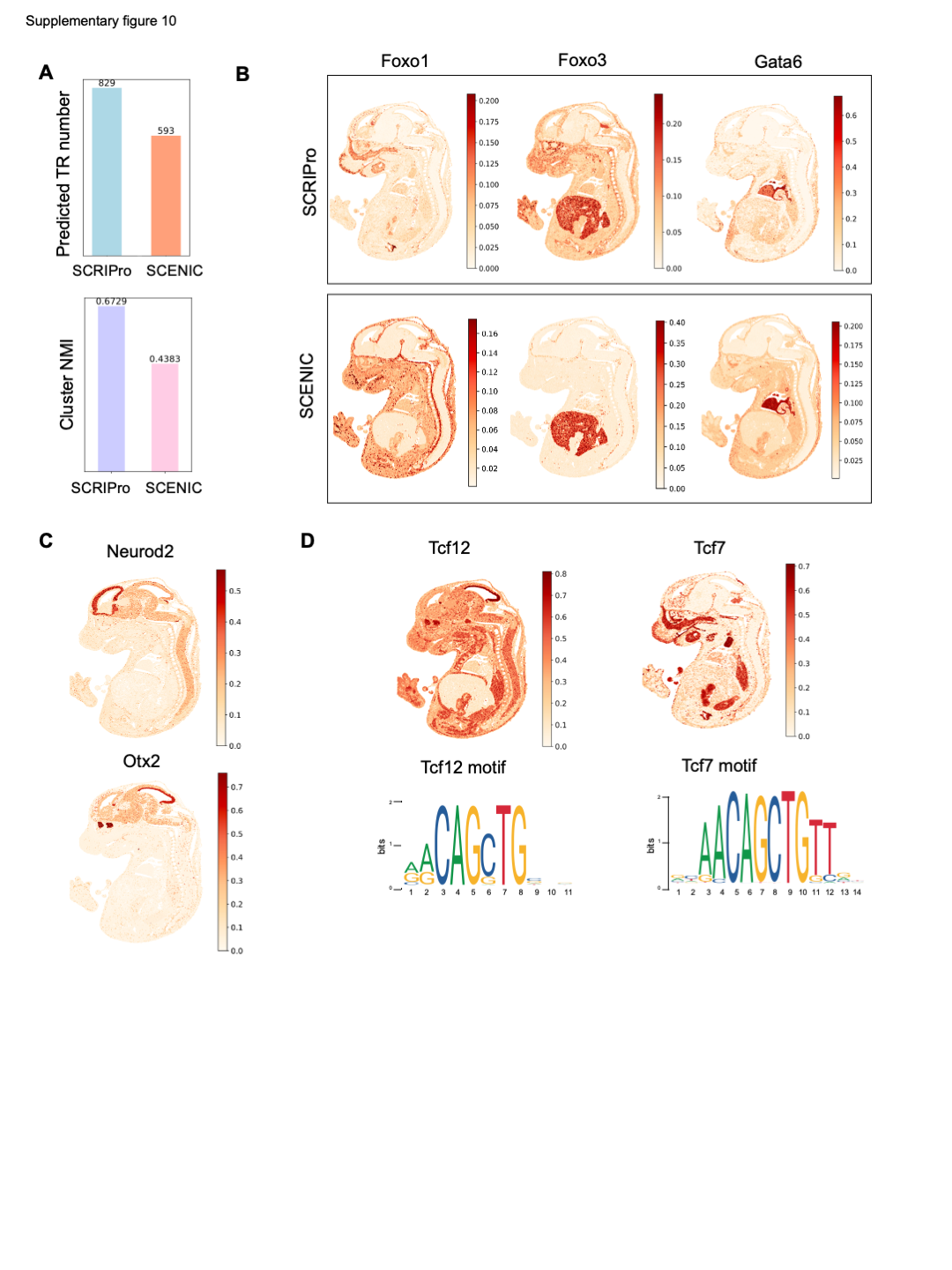


**Fig. S10 Spatial variable TRs co-expresssion modules and their distribution in E16.5 mouse embryo Stereo-seq dataset, Related to Fig.4**

1. Predicted TR number and cluster NMI score of SCRIPro and SCENIC.
2. Foxo1, Foxo3 and Gata6 predicted spatial distribution of SCRIPro and SCENIC.
3. TRs are predictable by SCRIPro but not in SCENIC, such as Neurod2 and Otx2.
4. Motif pattern and spatial distribution of Tcf12 and Tcf7.

**
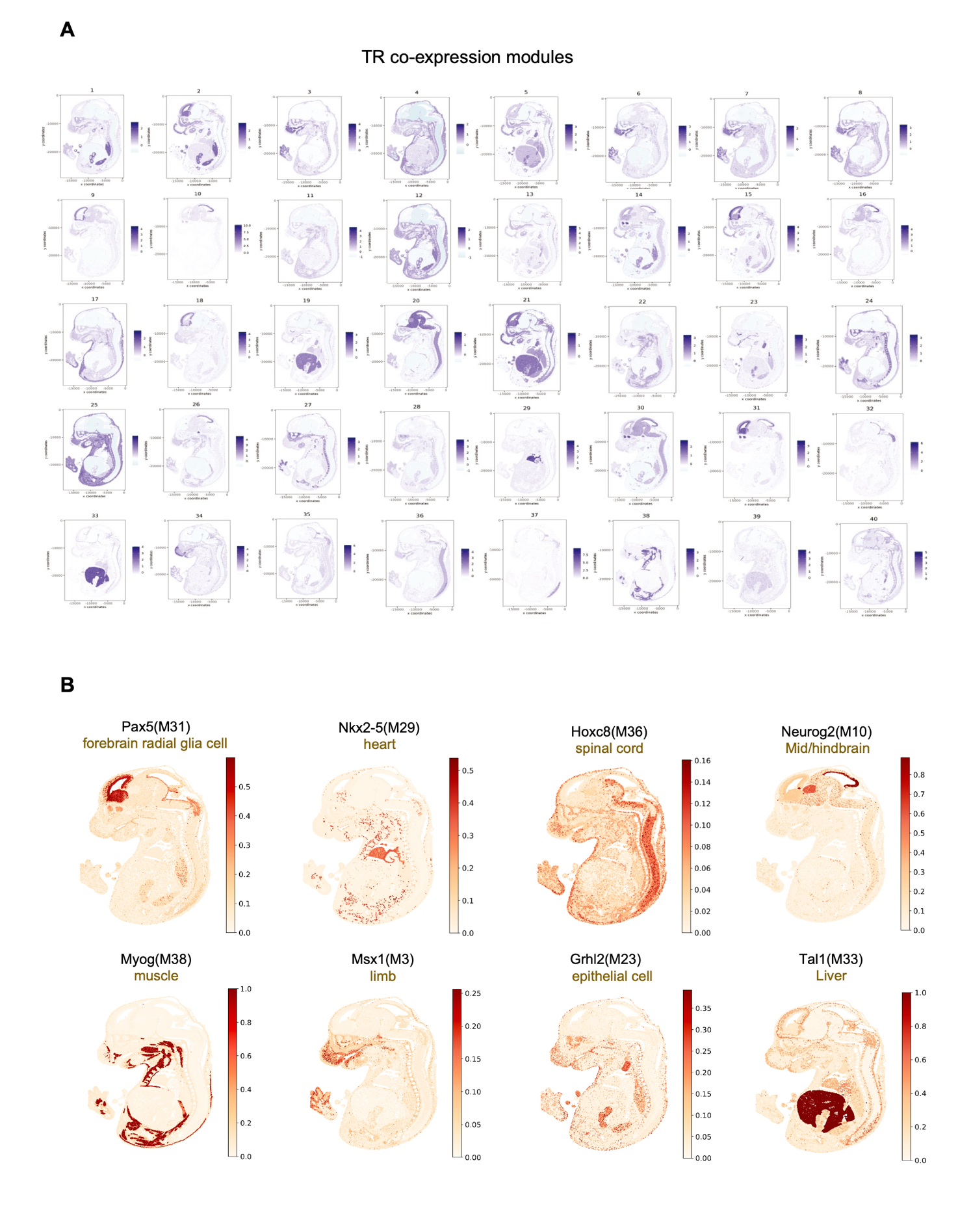
Fig. S11 Spatial variable TRs co-expresssion modules and their distribution in E16.5 mouse embryo Stereo-seq dataset, Related to Fig.4.**

1. 40 co-expression modules clustered by spatial variable TRs.
2. Examples of TR spatial modules are shown in each module.


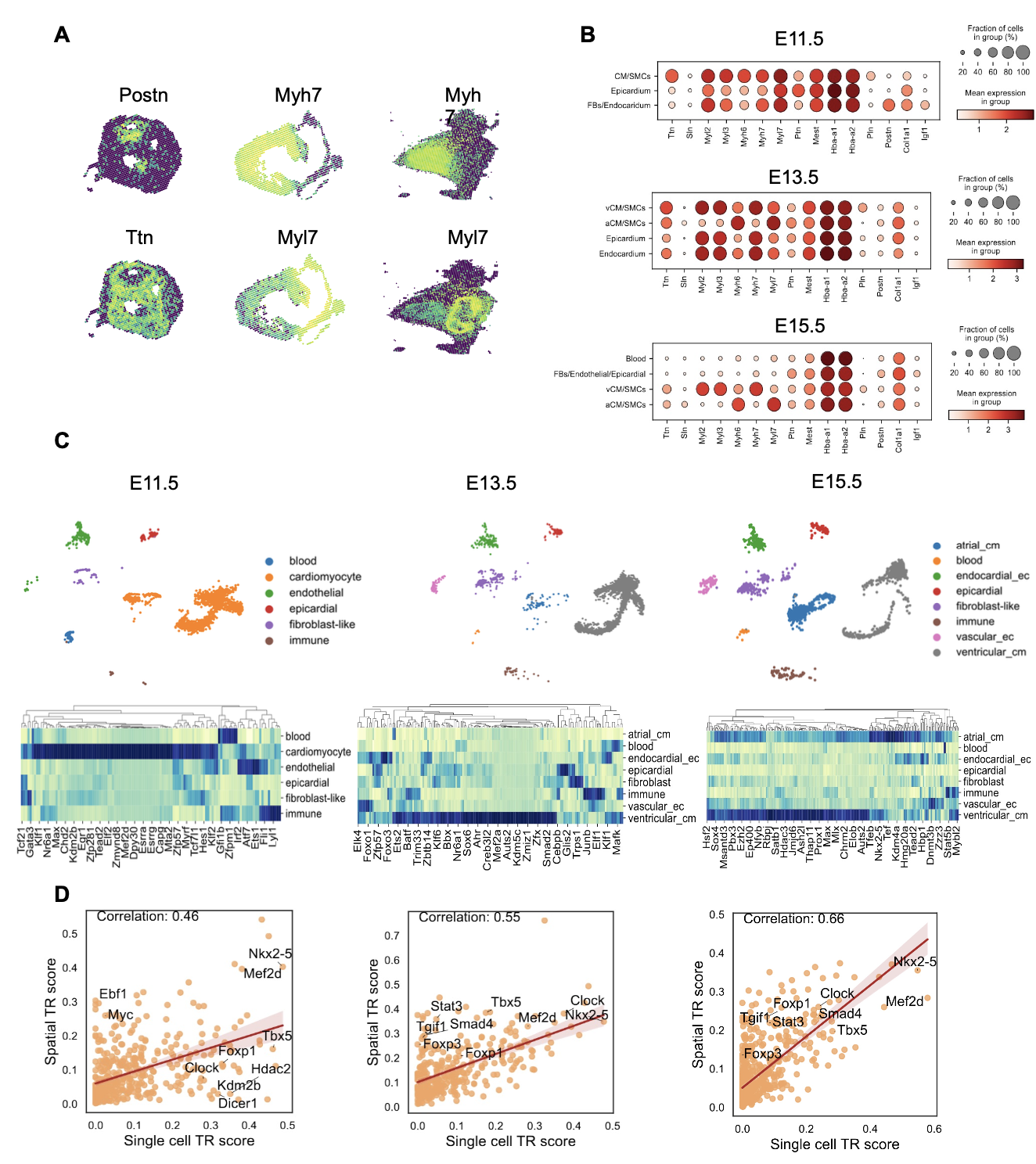
 **Fig. S12 Single cell mouse embryo across E11.5, E13.5 and E15.5 stages in embryonic hearts, Related to Fig.5.**

1. The spatial expression of epithelial marker gene *Postn*, cardiomyocyte marker gene *Ttn*, ventricular marker gene *Myh7*, and atrial marker gene *Myl7* are shown across three stages in the embryonic heart.
2. The dot plots of marker genes for each cell type across each developmental stage.
3. Single cell celltype UMAP clustering in E11.5, E13.5 and E15.5.
4. Spatial and single cell cardiomyocyte TRs correlation among three stages.


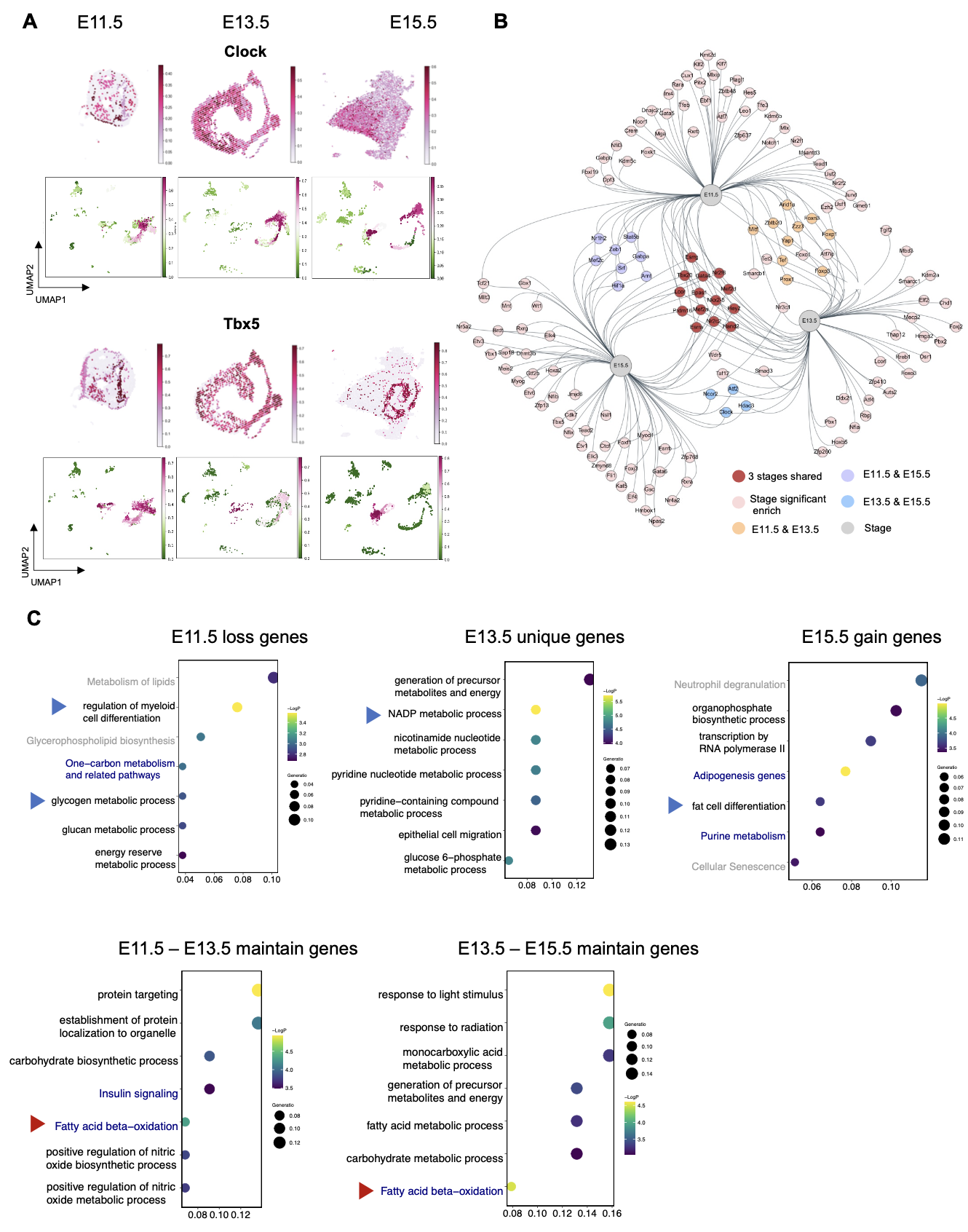
 **Fig. S13 PRDM16 target genes enrichment GO terms, Related to Fig.5.**

1. TR activity score distribution both in spatial and single cell of Clock and Tbx5 from E11.5 to E15.5.
2. Stage-specific TRs at E11.5, E13.5, and E15.5 developmental stages. Red dots: Significant TRs among three stages. Orange dots: TRs which are shared in E11.5 and E13.5 stages. Blue dots: TRs which are shared in E13.5 and E15.5 stages. Purple dots: TRs which are shared in E11.5 and E15.5 stages. Pink dots: TRs which are stage unique. Grey dots: Stage.
3. GO terms for target genes of PRDM16 during five distinct developmental stages: E11.5 loss, E13.5 unique, E15.5 gain, E11.5-E13.5 maintain, and E13.5-E15.5 maintain.

**
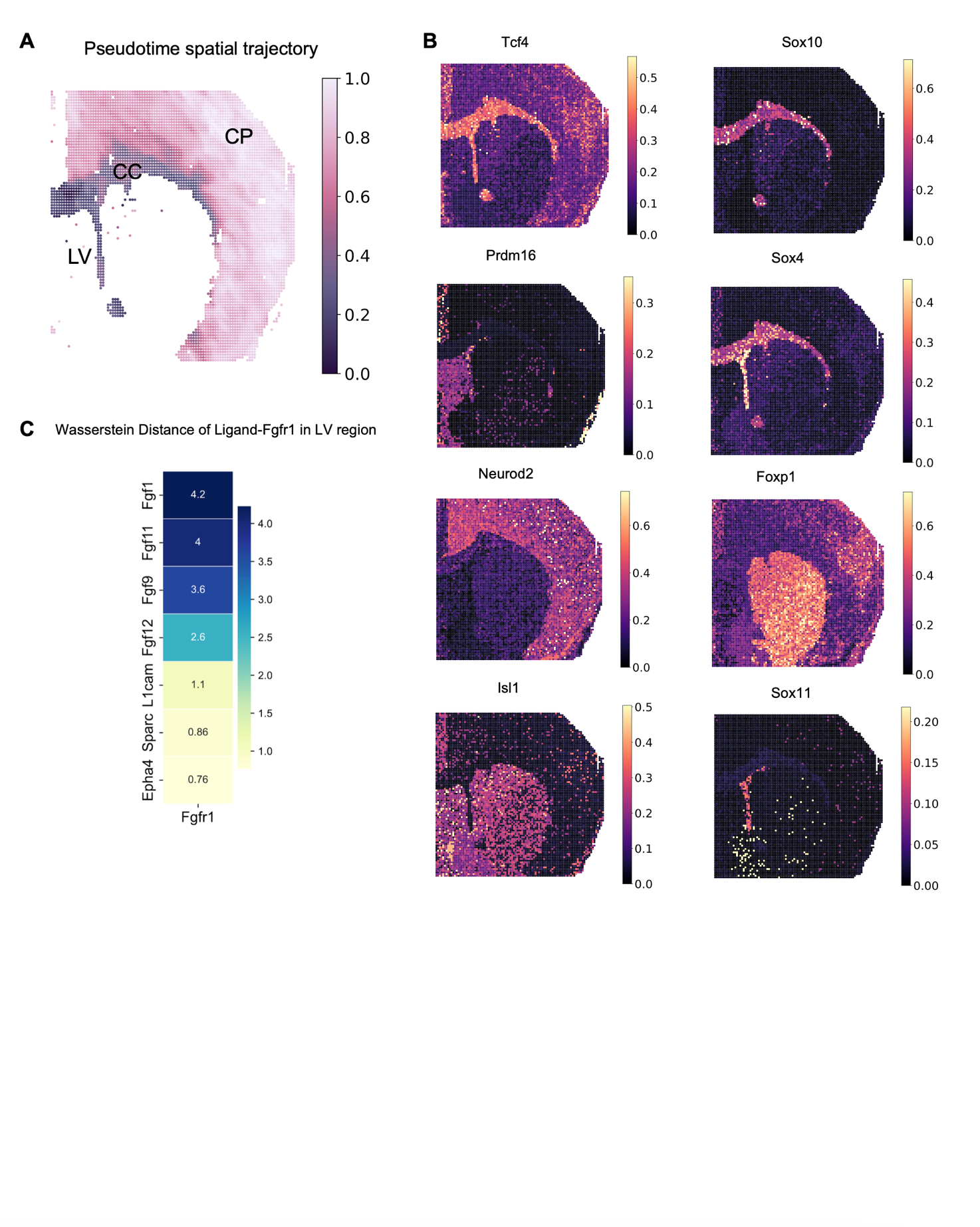
 Fig. S14 Cell type-specific TRs and L-R pathway in P22 mouse brain, Related to Fig.6.**

1. Pseudo time spatial trajectory analysis by SpaceFlow in LV, CC and CP region.
2. Examples of spatial variable TRs in each cell type.
3. Wasserstein Distance of Ligand-FGFR1 in LV region.
